# The NICE OECD countries' geographic search filters: Part 2—validation of the MEDLINE and Embase (Ovid) filters

**DOI:** 10.5195/jmla.2021.1224

**Published:** 2021-10-01

**Authors:** Lynda Ayiku, Thomas Hudson, Ceri Williams, Paul Levay, Catherine Jacob

**Affiliations:** 1 lynda.ayiku@nice.org.uk, Information Specialist, NICE Information Services team, National Institute for Health and Care Excellence, Manchester, UK; 2 thomas.hudson@nice.org.uk, Information Specialist, NICE Information Services team, National Institute for Health and Care Excellence, Manchester, UK; 3 ceri.williams@nice.org.uk, Information Specialist, NICE Information Services team, National Institute for Health and Care Excellence, Manchester, UK; 4 paul.levay@nice.org.uk, Information Specialist, NICE Information Services team, National Institute for Health and Care Excellence, Manchester, UK; 5 catherine.jacob@nice.org.uk, Information Specialist, NICE Information Services team, National Institute for Health and Care Excellence, Manchester, UK

**Keywords:** search filter, literature searching, geography

## Abstract

**Objective:**

We previously developed draft MEDLINE and Embase (Ovid) geographic search filters for Organisation for Economic Co-operation and Development (OECD) countries to assess their feasibility for finding evidence about the countries. Here, we describe the validation of these search filters.

**Methods:**

We identified OECD country references from thirty National Institute for Health and Care Excellence (NICE) guidelines to generate gold standard sets for MEDLINE (n=2,065) and Embase (n=2,023). We validated the filters by calculating their recall against these sets. We then applied the filters to existing search strategies for three OECD-focused NICE guideline reviews (NG103 on flu vaccination, NG140 on abortion care, and NG146 on workplace health) to calculate the filters' impact on the number needed to read (NNR) of the searches.

**Results:**

The filters both achieved 99.95% recall against the gold standard sets. Both filters achieved 100% recall for the three NICE guideline reviews. The MEDLINE filter reduced NNR from 256 to 232 for the NG103 review, from 38 to 27 for the NG140 review, and from 631 to 591 for the NG146 review. The Embase filter reduced NNR from 373 to 341 for the NG103 review, from 101 to 76 for the NG140 review, and from 989 to 925 for the NG146 review.

**Conclusion:**

The NICE OECD countries' search filters are the first validated filters for the countries. They can save time for research topics about OECD countries by finding the majority of evidence about OECD countries while reducing search result volumes in comparison to no filter use.

## INTRODUCTION

A search filter is a set of premade, reusable search terms with known performance characteristics [1, 2]. They are applied to literature search strategies to retrieve evidence for specific topics [1, 2]. Geographic search filters are applied to literature searches with the aim of retrieving evidence about geographic locations such as continents or countries [3].

Validating filters is important because it allows conclusions to be made about the generalizability of a search filter's performance [1, 2]. Validation provides users with an indication of how successfully filters work [2]. Validated search filters differ from search strategies because their recall (also known as “sensitivity”) has been established using a gold standard (GS) set (also known as a “reference set”) [1, 2]. Recall is the proportion of known, relevant results retrieved by a filter [1, 2].

Precision and number needed to read (NNR) are measures of search filter efficiency. Precision is the percentage of retrieved records that are relevant, and NNR is the number of records that must be screened to retrieve one relevant record [4]. Manually selecting references is a time-consuming (and therefore costly) aspect of systematic reviewing and guideline development [5]. In our experience at the United Kingdom's (UK) National Institute for Health and Care Excellence (NICE), even modest relative reductions in NNR can lead to worthwhile time savings. NICE guidelines provide evidence-based recommendations for preventing or managing specific conditions, planning services, and interventions to improve health and social care in the UK. Systematic literature searches are conducted to find the evidence for these guidelines.

### The Organisation for Economic Co-operation and Development

The Organisation for Economic Co-operation and Development (OECD) is an international agency that works in collaboration with high income, democratic countries to develop policies for reducing inequality and poverty in all nations [6]. There are currently thirty-eight OECD country members [6]. We previously drafted the NICE OECD countries' geographic search filters for MEDLINE and Embase (Ovid) [7] using our experience gained during the creation of the NICE UK geographic search filters [8, 9]. There is a nonvalidated filter search strategy available for OECD countries, but no validated filters previously existed [5].

In NICE guidelines, evidence about countries with similarities to the UK is often required. OECD countries are usually used as a proxy for similar countries. The purpose of creating the draft OECD filters was to assess their feasibility for finding evidence about OECD countries. The draft version of the filters used in the development study were promising, as they retained most OECD country evidence while reducing search result volumes [7].

### Final NICE OECD countries' geographic search filters

Following the development study, we updated and finalized the draft filters for the validation process [7]. The purpose of the current study was to validate the final version of the filters and evaluate their effectiveness and efficiency.

The NICE OECD countries' search filters take the unusual approach of finding evidence about OECD countries by excluding evidence about non-OECD countries. Detailed information about the structure and content of the filters is available in our previous paper [7]. Briefly, the filters are based on the commonly used “humans” search limit which was first appended to a search filter for systematic reviews [7, 10]. The humans limit excludes database records that only have subject headings for animals and retains all other database records to find database records about humans [7, 10].

The NICE OECD countries' search filters are composed of a set of subject headings for non-OECD countries that are applied to a separate set of OECD country subject headings with the NOT Boolean operator, as outlined below [7]:

1. Subject headings about non-OECD countries (combined with the OR Boolean operator)
2. Subject headings about OECD countries (combined with the OR Boolean operator)
3. 1 NOT 2

The filters must be applied to search strategies with the NOT Boolean operator, as in the following example [7]:

1. Search strategy
2. NICE OECD countries' search filter
3. 1 NOT 2

The filters work by excluding database records that have only the non-OECD subject headings, which are listed in the filters, and retaining all the remaining database records to find OECD country evidence. The filters retain database records that are indexed with only OECD country subject headings, that are indexed with both OECD and non-OECD country subject headings, and that are not indexed with either OECD or non-OECD country subject headings [7].

**Figure 1:**
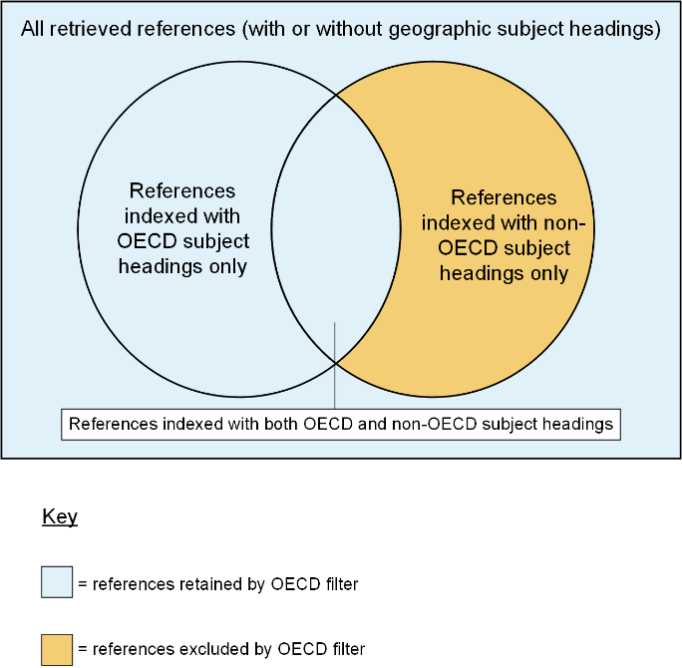
Venn diagram showing the conceptual structure of the OECD countries' filters (not to scale)

The Venn diagram in Figure 1, from our previous study [7], illustrates how the filters work.

Using the geographic subject headings to retrieve evidence directly would result in relevant OECD evidence being inadvertently missed. This is because some database records do not have any geographic subject headings. Because of this, we took a cautious approach of excluding unwanted geographic subject headings to retain relevant evidence in order to maximize the filters' retrieval.

As explained in our previous paper [7], creating a “traditional” search filter that includes free-text terms would be complex for finding evidence about several countries. For instance, a reasonably sensitive geographic filter that included free-text terms would likely need to include free-text search terms for countries and all city names for each country, free-text search terms for the national or regional health services of each country, and relevant language variations for all countries, cities, and health services [3, 7]. The NICE OECD countries' filters do not include free-text terms to lower the risk of inadvertently excluding relevant geographic evidence.

### Aim and objectives

The aim of the NICE OECD countries' search filters is to improve the effectiveness and efficiency of literature searches when evidence about OECD countries is required. Our objectives were 1) to create novel validated OECD countries search filters that retrieve evidence about OECD countries from MEDLINE and Embase (Ovid) with high recall and 2) to reduce NNR in comparison to no filter use. The target for recall was set at 90% in line with previous filter studies [11].

## METHODS

### Generation of the gold standard sets

Search filters are validated using a gold standard (GS) set of known, relevant references [1, 2]. The relative recall method approach was used to identify references about OECD countries to form the GS sets for the filters [12]. The method involves pooling relevant references that have informed several evidence reviews to calculate the recall of search filters [12].

We identified the OECD country-related references for the MEDLINE and Embase GS sets from the evidence reviews of thirty NICE guidelines that were published between December 2018 and December 2019 (see Appendix 1 for the NICE guidelines).

We screened the evidence tables for the reviews that informed each NICE guideline to identify references that had been categorized with an OECD country or a group of countries that included at least one OECD country. There were too many unique OECD country references to list them in a single search string [13]. Instead, we created individual search strings for the thirty-two OECD countries for which we found references and a separate search string for the references that reported studies taking place in multiple countries. This ensured there were no duplicate records in our gold standard set (see Appendix 2 for the number of references found for each OECD country). Each search string contained the primary author, title, and publication date for each OECD country reference on individual search lines. We combined the search string lines with the OR Boolean operator at the end (see Appendix 3 for an example of these search strings). We saved the thirty-three search strings so that they could be rerun to validate the filters.

We found 2,065 references that were available in MEDLINE (see Appendix 4) and 2,023 references that were available in Embase (see Appendix 5). We used these references to form the GS sets for each database.

A sample size of at least 100 relevant references is suggested to provide a reasonable confidence interval for a filter that aims to retrieve at least 90% of all relevant references [12]. Both the MEDLINE and Embase validation sets exceeded this minimum specification.

### Validating the NICE OECD countries' filters

We validated the filters by calculating their recall against their GS sets. To do this, we applied the MEDLINE and Embase filters to each of the thirty-three saved OECD country search strings in turn using the NOT Boolean operator (see Appendix 3 for an example). We recorded the recall results individually and then added them together to form an overall recall result, given that there were no duplicates in the GS (see Appendix 2). Recall was calculated as (No. of GS set references retrieved by search filter/Total no. of GS set references) × 100 to express as a percentage [2].

### Case study: Evaluating the filters' efficiency using NNR

After the filters were validated, we conducted a case study to evaluate the efficiency of the filters using “real life” searches. The filters had been validated for sensitivity using lists of individual references from thirty different guidelines. This did not demonstrate their impact on reducing the screening workload for search topics.

For the case study, we used the original MEDLINE and Embase search strategies from three NICE guideline evidence reviews that were based on OECD country evidence. No geographic restrictions had been applied to the original search strategies. The three NICE guideline evidence reviews topics that we used are below:

- NG103 Flu vaccination: increasing uptake.
  - Evidence review 3: Increasing uptake in clinical risk groups [14]
- NG140 Abortion care.
  - Evidence review P: Contraception after abortion [15]
- NG146 Workplace health: Long-term sickness absence and capability to work.
  - Evidence review C: Facilitating the return to work of employees on long-term sickness absence and reducing risk of recurrence [16]

We reran the original Embase searches on December 15, 2020, and reran the original MEDLINE searches on December 18, 2020. The rerun search strategies contained date limits that matched the time frame of the original search strategies. We calculated the original search strategies' recall and NNR for retrieving the included references for the reviews. We then applied the OECD countries' filters to the strategies using the NOT Boolean operator and compared the recall and NNR of the filtered strategies for finding the included references. Recall was calculated as (No. of included references retrieved by search filter/Total no. of included references) × 100 to express as a percentage [2]. NNR was calculated as 1/precision [4], with precision equal to the proportion of references retrieved by a filter that are relevant: (No. of relevant included references retrieved by search/Total no. of references retrieved by the search) [2].

## RESULTS

The validated NICE OECD countries' geographic search filters for MEDLINE and Embase can be found in Appendix 6.

### NICE OECD countries' filters results

We validated the MEDLINE and Embase filters by calculating their recall against the MEDLINE and Embase GS set references. The filters both met the >90% target for high recall. Both filters achieved 99.95% recall (2,064 out of 2,065 MEDLINE GS set references and 2,022 out of 2,023 Embase GS set references) (Table 1).

**Table 1:** Validation of the MEDLINE and Embase OECD countries' filters

Database	No. of gold standard references	No. of gold standard references retrieved by filter	Recall
MEDLINE	2,065	2,064	99.95%
Embase	2,023	2,022	99.95%

The filters both missed one reference that concerned OECD and non-OECD countries [17, 18]. The reference missed by the MEDLINE filter was about a model developed by researchers based in both OECD and non-OECD countries for predicting adverse maternal outcomes in pregnancy hypertension in low and middle income countries (LMICs) [17]. The reference missed by the Embase filter was about the same model for assessing and triaging women with hypertensive disorders of pregnancy in low-resourced settings [18].

### Case study

In the case study, the versions of the search strategies with the filters applied achieved 100% recall for the included references that were found by the original search strategies (Table 2).

**Table 2:** Case study results

	Original search strategy			Filtered search strategy			
	No. of search hits retrieved	No. of included references retrieved	NNR	No. of search hits retrieved	No. of included references retrieved	Recall of included references found by original strategy	NNR
**NG103: Evidence review 3: increasing uptake in clinical risk groups**							
MEDLINE	5,635	22	256	5,108	22	100%	232
Embase	9,707	26	373	8,884	26	100%	341
**NG140: Evidence review P: contraception after abortion**							
MEDLINE	620	16	38	443	16	100%	27
Embase	1619	16	101	1216	16	100%	76
**NG146: Evidence review C: facilitating the return to work of employees on long-term sickness absence and reducing risk of recurrence**							
MEDLINE	13,902	22	631	13,022	22	100%	591
Embase	20,775	21	989	19,443	21	100%	925
**NG103, NG140, and NG146 combined**							
MEDLINE	20,157	60	336	18,573	60	100%	309
Embase	32,101	63	510	29,543	63	100%	469

For the NG103 evidence review, the MEDLINE filter reduced NNR from 256 to 232, and the Embase filter reduced NNR from 373 to 341. For the NG140 evidence review, the MEDLINE filter reduced NNR from 38 to 27, and the Embase filter reduced NNR from 101 to 76. For the NG146 evidence review, the MEDLINE filter reduced NNR from 631 to 591, and the Embase filter reduced NNR from 989 to 925. On average the filters reduced the volume of references to be screened by 8% (Table 2).

The search strategies and search result volumes for the MEDLINE filter can be found in Appendix 7. The details for the Embase filter can be found in Appendix 8.

## DISCUSSION

The NICE OECD countries' geographic search filters for MEDLINE and Embase are the first validated filters to find evidence about OECD countries. To our knowledge, the filters are also the first validated, standalone search filters to be composed entirely of subject headings. In addition, the filters are the first validated, standalone filters to make exclusive use of the NOT Boolean operator with the aim of excluding irrelevant evidence to retain relevant evidence.

The filters found almost all the OECD country evidence in the NICE evidence reviews from MEDLINE and Embase while reducing search result volumes by a modest but useful amount. The Cochrane Handbook estimates that 500–1,000 abstracts can be screened in an eight-hour period [19]. The 8% reduction in search volumes that we found would be equivalent to a day reclaimed for each twelve to thirteen days spent screening. Given the numbers of reviews conducted within our own organization that focus on OECD countries, this adds up to a significant productivity gain compared to the current practice of running searches without geographic filters and manually rejecting more non-OECD references.

We acknowledge that databases additional to MEDLINE and Embase are used for systematic literature searches [19]. Although it is possible for the filters to be translated for other databases that have geographic subject headings, it is important to note that the translated filters would not be validated. Unfortunately, the filters cannot be used for databases without geographic subject headings. However, as MEDLINE and Embase are typically the largest health databases that are used for systematic literature searches, it is likely that overall search result volumes will be reduced if the filters are used in MEDLINE and Embase.

The principles behind the filters can potentially be used to find evidence for a variety of other country groupings. For example, for research topics that require evidence about BRICS (Brazil, Russia, India, China, South Africa) [20] or World Bank countries [21], searchers could transfer subject headings from the non-OECD countries section of the filter to the OECD countries section to retain the additional relevant geographic results. Similarly, the geographic subject heading sets for OECD and non-OECD countries in the filters could be reversed. This would exclude search results about high-income nations for research topics about LMICs. We are currently exploring the use of modified versions of the filters for finding evidence about other country groups.

There are currently only three validated geographic search filters for the UK, Africa, and Spain in the published literature [3, 8–9, 22–24]. We are also aware of a forthcoming publication about a validated filter for the United States [25]. We hope that our work on geographic search filters demonstrates their usefulness and will encourage the development of additional validated search filters for more locations around the world [3].

### Limitations

As detailed in our previous paper on the development of the filters [7], we acknowledge that using geographic subject heading terms alone for the filters has limitations. One limitation is that not all references with clearly defined geographic settings in other search fields are indexed with geographic subject headings [5]. The disadvantage is that irrelevant non-OECD country evidence is retained in these cases. However, this limitation does not pose risks for OECD evidence to be excluded inadvertently.

As in the development study [7], the validated filters did not exclude any database records that were focused solely on an OECD country. However, we suggest that the filters should be used with caution for topics that concern both OECD and non-OECD countries (for example, international adoption or international migration). This is because some database records for topics about both OECD and non-OECD countries only have geographic subject headings for the non-OECD countries. This can result in OECD evidence being excluded inadvertently. For example, the validated filters missed a total of two references that concerned both OECD and non-OECD countries (i.e., LMICs) [17, 18], as the database records for the references only had subject headings for the LMICs. A similar finding was seen in the development study [7].

In our previous study, we noted that the geographic search filters may need to be updated to reflect changes in OECD membership, MeSH, and Emtree OECD and non-OECD country terms as well as changes to the names of the countries [7]. However, as the filters are composed of subject headings, updating the filters should be straightforward.

## CONCLUSION

The NICE OECD countries' search filters for MEDLINE and Embase are the first validated filters to find evidence about OECD countries. The filters find the majority of evidence about OECD countries while reliably reducing search result volumes in comparison to no filter use. They can therefore potentially save time for research topics about OECD countries.

## Data Availability

Data associated with this article (Appendixes 1–8) are available at: https://osf.io/k7tcf/
